# Mechanical and Sustained-Release Properties of Crosslinked Poly(vinyl alcohol)/Sodium Humate Composite Membranes

**DOI:** 10.3390/polym18172045

**Published:** 2026-08-23

**Authors:** Shuai Kuang, Enwei Chen, Tian-en Shui, Piyue Gong, Feng Wang, Haiying Huang

**Affiliations:** 1 State Key Laboratory of Advanced Optical Polymer and Manufacturing Technology, Key Laboratory of Rubber-Plastics, Ministry of Education, School of Polymer Science and Engineering, Qingdao University of Science and Technology, Qingdao 266042, China; 2 State Key Laboratory of Polymer Science and Technology, Changchun Institute of Applied Chemistry, Chinese Academy of Sciences, Changchun 130022, China

**Keywords:** sodium humate, polyvinyl alcohol, composite membrane, crosslinked networks, mechanical properties, sustained-release

## Abstract

Humic acid, as a natural macromolecular aggregate rich in functional groups, offers abundant modification sites and tunable chemical functionality, making it a promising building block for three-dimensional network construction. In this study, glutaraldehyde (GA) was employed as a crosslinking agent to incorporate sodium humate (NaHA, sodium salt of humic acid from alkaline treatment) into a polyvinyl alcohol (PVA) matrix, yielding composite membranes with enhanced structural stability and performance. The results demonstrate that NaHA effectively modulates the crosslinked network, and the physical and mechanical characteristics can be readily tailored by varying the PVA/NaHA/GA ratio. Compared with pristine PVA/GA hydrogel, the inclusion of NaHA significantly influences the mechanical response, with optimal comprehensive performance achieved at a NaHA content of 7.5 wt%, corresponding to a tensile strength of 60.4 MPa and an elongation at break of 74.2%. Furthermore, the cumulative release of NaHA after two days reached 64.4%, confirming that NaHA supramolecular aggregates were stably entrapped within the PVA/GA crosslinked matrix. The release kinetics were well described by the Korsmeyer–Peppas model. Overall, the covalent crosslinking of PVA with GA, together with hydrogen-bonding associations and physical entrapment mediated by NaHA, constructed a composite membrane network. This network exhibited tunable dry-state mechanical properties and sustained NaHA release, supporting its further evaluation as a prospective candidate for agricultural mulching films.

## 1. Introduction

Humic acid (HA) is a major component of Earth’s organic carbon, with surface soil stores exceeding combined carbon in the biosphere and atmosphere [1]. As a polyanionic weak acid, HA has a heterogeneous structure rich in oxygen-containing groups (e.g., hydroxyl, carboxyl, carbonyl, amino), which enable functions like soil aggregation, plant growth promotion, and improved fertilizer efficiency [2,3,4,5]. Unlike synthetic polymers, HA lacks repeating units and has a broad molecular weight distribution, leading to variable properties depending on source and extraction process [6]. This complexity has restricted HA use mainly to soil conditioning and plant growth stimulation, leaving its potential in functional materials largely unrealized [7,8]. Meanwhile, sustainable agriculture demands advanced polymeric materials. Plastic films and seedling substrates can improve soil temperature, moisture retention, weed suppression, and crop yield; however, conventional petroleum-based plastics, such as polyethylene, cause persistent “white pollution.” [9,10]. Thus, developing biodegradable materials with agronomic benefits (e.g., nutrient release, soil improvement) is a key challenge at the intersection of polymer science and agriculture [11].

From the perspective of molecular design, humic acid provides a unique opportunity to address these challenges. The abundant reactive functional groups within its molecular framework can participate in graft copolymerization and chemical crosslinking reactions, while simultaneously forming physically crosslinked networks through noncovalent interactions, such as hydrogen bonding, with polar groups on synthetic polymer chains [12,13,14]. The resulting hybrid materials possess three-dimensional network architectures that are, in principle, capable of integrating multiple functionalities, including metal-ion complexation, water regulation, and controlled fertilizer release. Nevertheless, a fundamental dilemma has hindered further progress in this field. Although HA is inherently hydrophilic, water absorption and swelling can readily disrupt the weak intermolecular interactions that maintain its physical structure, leading to pronounced deterioration in dimensional stability and mechanical integrity. Consequently, HA-based materials often fail to meet the durability requirements demanded by practical agricultural and environmental applications [5,15].

To date, research on HA-based hydrogels has largely focused on noncovalent fabrication strategies, such as physical blending or simple encapsulation, in which HA is typically incorporated through physical doping with monomers (e.g., acrylic acid) or serves as an inert filler within interpenetrating polymer networks. As a result, the intrinsic capacity of HA to act as a versatile macromolecular crosslinker and a functional framework component remains largely underexplored [16,17,18,19,20,21,22]. For example, Sirousazar et al. prepared HA/poly(vinyl alcohol) supramolecular hydrogels using repeated freeze–thaw cycles and demonstrated that HA nanoparticles could act as physical crosslinking sites by promoting the aggregation of PVA chains. However, because the network relied primarily on noncovalent interactions, such as hydrogen bonding, its structural stability under humid or aqueous conditions remained limited [23]. Khatun et al. fabricated PVA/sodium alginate/HA composite hydrogels using citric acid as a chemical crosslinker and achieved preliminary controlled-release behavior for urea fertilizer. Nevertheless, HA functioned mainly as a filler rather than a structural constituent of the polymer network, overlooking its potential participation in crosslinking interactions [24]. More recently, Torres-Figueroa et al. systematically compared physically and chemically crosslinked PVA/HA composite hydrogels and showed that HA could enhance network strength through both physical interactions and modulation of chemically crosslinked systems. Their findings reveal that humic acid is not merely an inert filler but actively reinforces the network via physical interactions while also modulating the chemical crosslinking system [25].

Concurrently, the molecular engineering of chemically crosslinked HA-based networks suffers from a notable absence of systematic theoretical frameworks. In particular, the quantitative structure–property relationships linking crosslinking parameters, such as crosslinking density and network homogeneity, to macroscopic properties including water uptake, water retention, and mechanical performance remain poorly understood. This knowledge gap has hindered both the rational design of high-performance HA-based sustainable materials and a fundamental understanding of the intrinsic relationship between molecular structure, network architecture, and material properties. Such challenges are further amplified when transitioning from loosely crosslinked hydrogels to dense film architectures, where higher mechanical integrity and structural uniformity are required.

Chemical crosslinking enables the formation of robust three-dimensional polymer networks with high mechanical strength, excellent solvent resistance, and well-defined network architectures. Driven by prospective agricultural applications that demand both film processability and controlled solute release, this study focuses on dry composite membranes based on polyvinyl alcohol (PVA) and sodium humate (NaHA), formed via GA-mediated crosslinking of PVA. Sodium humate is the sodium salt obtained from the alkaline treatment of humic acid; it exhibits significantly improved aqueous solubility and a weak alkaline pH (8~9), which substantially broadens its applicability as a functional polymeric material. Herein, we examine whether the incorporation of NaHA into PVA facilitates the formation of self-assembled composite membranes that synergistically combine structural stability with robust mechanical performance. Furthermore, we elucidate how the physicochemical and mechanical properties of the resultant membranes evolve with varying NaHA contents and GA loading and assess the sustainability of NaHA release under environmentally relevant conditions. Collectively, this work offers a molecular engineering strategy for the design of eco-friendly multifunctional composite membranes tailored for sustainable agricultural systems.

## 2. Materials and Methods

### 2.1. Materials

Poly(vinyl alcohol) (PVA, Mn = 131.5 kDa, PDI = 1.2, degree of hydrolysis: 98~100%) was purchased from Polymer Source Inc. (Montreal, QC, Canada). Sodium humate (NaHA) was obtained from Aladdin Biochemical Technology Co., Ltd. (Shanghai, China) and purified prior to use. Glutaraldehyde (GA, 50 wt% aqueous solution), used as a chemical crosslinker, was purchased from Sinopharm Chemical Reagent Co., Ltd. (Shanghai, China). Deionized (DI) water with a resistivity of 18.2 MΩ·cm, produced by a Millipore Milli-Q system, was employed in all preparation and testing procedures.

To obtain purified NaHA, the as-received crude powder was first dissolved in 60 mL of DI water, and the resulting solution was centrifuged at 12,000 rpm for 30 min. The collected supernatant was then dialyzed against DI water for 2 days, with frequent water changes, to remove low-molecular-weight impurities. After dialysis, the retentate was rapidly frozen in liquid nitrogen and subsequently lyophilized to yield purified NaHA as a black powder. All other reagents were used as received without further purification.

### 2.2. Preparation of Composite Membranes

A series of composite membranes were fabricated via a facile solution casting method. Initially, a 5 wt% working solution of glutaraldehyde (GA) was prepared gravimetrically by diluting a commercial 50 wt% aqueous GA stock solution with deionized (DI) water. Separately, 5 g of PVA was completely dissolved in 100 mL of DI water under constant magnetic stirring at 90 °C in an oil bath for 4 h, resulting in a homogeneous and transparent polymer solution. The system was then cooled to 65 °C, and a predetermined amount of purified NaHA was added. The pH of the mixture was adjusted to 4.0 using acetic acid as the sole acidifying agent. To ensure uniform dispersion of NaHA, the mixture was continuously stirred in the dark for an additional 4 h. After naturally cooling to ambient temperature, a calculated volume of the 5 wt% GA working solution was introduced dropwise, and the mixture was stirred for 1 h. Subsequently, the solution was ultrasonicated for 30 min to remove entrapped air bubbles. An aliquot of 30 mL of the resulting membrane-forming solution was then accurately transferred into a horizontal polytetrafluoroethylene (PTFE) mold with a diameter of 90 mm. The solution was dried in a forced-air oven at 45 °C for 24 h until a constant weight was achieved. The resulting membranes were carefully peeled from the mold and stored over color-indicating silica gel in a desiccator for at least 48 h prior to characterization.

To enable bidirectional orthogonal validation, two formulation series were prepared, as summarized in Table 1. In Series 1, the volume of the 5 wt% GA working solution (designated as V_GA_) was fixed at 5.0 mL, whereas the NaHA content, expressed as a percentage of the dry mass of PVA, was varied across 0, 5, 7.5, 10, 12.5, and 15 wt%. In Series 2, the NaHA content was held constant at 7.5 wt%, while the GA loading volume, V_GA_, was adjusted to 0, 2.5, 5.0, 7.5, and 10.0 mL. It should be noted that the suffix in the V_GA_ notation indicates the loading volume of the working solution in milliliters and does not represent the final GA weight percentage in the complete formulation.

### 2.3. Sustained Release of Composite Membranes

The sustained-release behavior of NaHA from the composite membranes was evaluated via a direct immersion method. A calibration curve was established using NaHA standard solutions at concentrations of 10, 50, 100, and 200 μmol/L (equivalent to 2.261, 11.307, 22.614, and 45.228 mg/L based on the labeled molar mass of 226.14 g/mol), fitted as *C* = (*Abs* − *b*)/*k,* where *Abs* denotes the measured absorbance. The dried composite membranes were cut into 2 cm × 2 cm specimens, weighed (*m*_0_, 0.1 ± 0.01 g), and immersed in 50 mL of deionized water at room temperature. At designated intervals (0.5, 1, 2, 4, and 8 h), aliquots were sampled for UV-Vis analysis and immediately returned to the release vessel, with the release volume maintained at 0.050 L throughout the experiment. Absorbance readings were converted to real-time concentrations, *C_n_* (mg/mL), using the calibration curve. The cumulative released mass (*M_n_*, mg) at each time point was calculated via Equation (1). For the PVA/NaHA_7.5%_-V_GA5_ formulation, the initial NaHA fraction was corrected to 6.67% on a dry-feed basis (including estimated GA solids), and the corresponding nominal loading (*M_total_*) was obtained from Equation (2). The cumulative release rate (*R*, %) was then determined using Equation (3).

*M_n_* = *C_n_* × 0.05L (1)

*M_total_* = *m*_0_ × 6.67% (2)

(3) $$R=\frac{M_{n}}{M_{\mathrm{total}}}\times 100\%$$

To derive an order-of-magnitude estimate of the structural characteristic length scale for the release formulation, the average effective mesh size of the PVA/NaHA_7.5%_-V_GA5_ composite membrane was determined from equilibrium swelling data using the classical Flory–Rehner theory [26]. Specimens of known dry mass (*W_d_*) were immersed in deionized water at 25 °C until a constant swollen mass (*W_s_*) was attained. The equilibrium polymer volume fraction ($\upsilon_{2,s}$) was subsequently calculated according to Equation (4) [27,28,29]:

(4) $$\upsilon_{2,s}={\left[1+\frac{\rho_{p}}{\rho_{w}}\left(\frac{W_{s}}{W_{d}}-1\right)\right]}^{-1}$$

where $\rho_{p}=1.26$ g/cm^3^ and $\rho_{w}=1.00$ g/cm^3^. The effective crosslinking density ($\nu_{e}$) and the model-derived average effective mesh size (ξ) were derived from Equations (5) and (6), respectively:

(5) $$\nu_{e}\;=\;-\frac{\ln\left(1-\upsilon_{2,s}\right){+\upsilon }_{2,s}{+\chi \upsilon }_{2,s}^{2}}{V_{1}\left(\upsilon_{2,s}^{1/3}-\frac{\upsilon_{2,s}}{2}\right)}$$

(6) $${\xi =\upsilon }_{2,s}^{-1/3}\cdot l\cdot \sqrt{\frac{{2C}_{n}\left(\frac{\rho_{p}}{\nu_{e}}\right)}{M_{r}}}$$

where χ represents the Flory–Huggins interaction parameter between the polymer and the solvent (approximately 0.49 at room temperature); *V*_1_ is the molar volume of water (18 cm^3^/mol); *l* is the average bond length of the main chain C-C bond (0.154 nm); *C_n_* is the Flory characteristic ratio describing the flexibility of the polymer chain (set as 8.3); *M_c_* corresponds to the average molecular weight between crosslinking points ($M_{c}{\;=\;\rho }_{p}/\nu_{e}$); and *M_r_* is the relative molecular mass of the repeating structural unit of PVA (44 g/mol).

### 2.4. Characterization of Composite Membranes

The chemical structural evolution and crosslinked network formation within the composite membranes were characterized via Fourier-transform infrared (FTIR) spectroscopy (INVENIO-R, Bruker, Ettlingen, Germany) equipped with an attenuated total reflectance (ATR) accessory. The spectra were recorded over a scanning wavenumber range of 4000–400 cm^−1^ with a resolution of 4 cm^−1^ and 128 cumulative scans.

X-ray diffraction (XRD) patterns were recorded on a D8 ADVANCE diffractometer (Bruker, Ettlingen, Germany) over a 2θ range of 5~40° at a scanning rate of 2°/min. All patterns were normalized to the maximum intensity of the respective diffractogram.

The cross-sectional fracture surfaces of the composite membranes were examined using a field-emission scanning electron microscope (SEM, Sigma 300, Zeiss, Oberkochen, Germany) at an accelerating voltage of 5 kV and a working distance of approximately 6.6 mm. Prior to imaging, the specimens were brittle-fractured in liquid nitrogen and sputter-coated with a thin layer of gold to enhance conductivity.

Mechanical properties were assessed in accordance with the Standard GB/T 1040.2-2022 [30] using a universal testing machine (INSTRON 5869, Norwood, MA, USA). All test specimens were prepared with the same dumbbell-shaped cutting die, featuring a reduced section of 16 mm in length and 4 mm in width. The thickness of the specimens, used for stress calculation, varied between 0.07 and 0.12 mm. For each formulation, three specimens were tested (*n* = 3) at room temperature with a crosshead speed of 50 mm/min. The relative humidity measured during testing was 21% under ambient conditions, not a controlled preconditioning environment. Tensile strength and elongation at break were calculated and are presented as the mean ± standard deviation for each set of three replicates.

Static water contact angle (WCA) measurements were performed using an optical contact angle goniometer (DSA30, KRÜSS GmbH, Hamburg, Germany). A 2 μL droplet of deionized water was gently deposited onto the membrane surface, and the droplet profile was captured within 1 s. For each sample, at least three distinct positions were measured to obtain an averaged value, minimizing potential errors arising from surface heterogeneity.

The release kinetics of NaHA from the composite membranes were quantitatively investigated using a UV-Vis spectrophotometer (UV-765, Shanghai Precision & Scientific Instrument Co., Ltd., Shanghai, China). Absorbance measurements were collected within the characteristic absorption range of NaHA (214–238 nm). A calibration curve relating absorbance to NaHA concentration was established, and linear regression analysis was applied to derive the release profiles.

## 3. Results and Discussion

Poly(vinyl alcohol) (PVA) is an excellent matrix material for hydrogel membranes owing to its outstanding film-forming capability and versatile crosslinking chemistry. On one hand, the hydroxyl groups on PVA chains readily undergo acetalization reactions with aldehyde crosslinkers, such as formaldehyde and glutaraldehyde, to form stable three-dimensional covalent networks, thereby substantially enhancing the structural integrity and chemical stability of the hydrogel [31,32]. On the other hand, blending PVA with functional components, including poly(acrylic acid), starch, and lignin, has been demonstrated to effectively tailor the microstructure of the polymer network, leading to simultaneous improvements in mechanical strength and toughness [33,34,35,36,37]. Building upon these advantages, sodium humate (NaHA) was incorporated into the PVA matrix as a multifunctional component to fabricate composite membranes. During the crosslinking process, NaHA was integrated into the PVA acetal network through intermolecular interactions, primarily hydrogen bonding, thereby participating in the formation of the three-dimensional polymer network.

To elucidate the structure–property relationships of the resulting composite membranes, this study was conducted in three sequential steps. First, Fourier transform infrared (FTIR) spectroscopy and X-ray diffraction (XRD) were employed to characterize spectroscopic interactions and provide qualitative insight into the structural features. Second, the influence of key formulation variables, namely, NaHA content and GA loading, on the mechanical properties (tensile strength and elongation at break) and surface wettability of the composite membranes was systematically examined. Finally, the cumulative release of NaHA from the membranes was quantified via UV–visible spectroscopy to assess the sustained-release profile and to delineate the corresponding release kinetics.

### 3.1. Crosslinking Networks in PVA/NaHA/GA Composite Membranes

**Figure 1:**
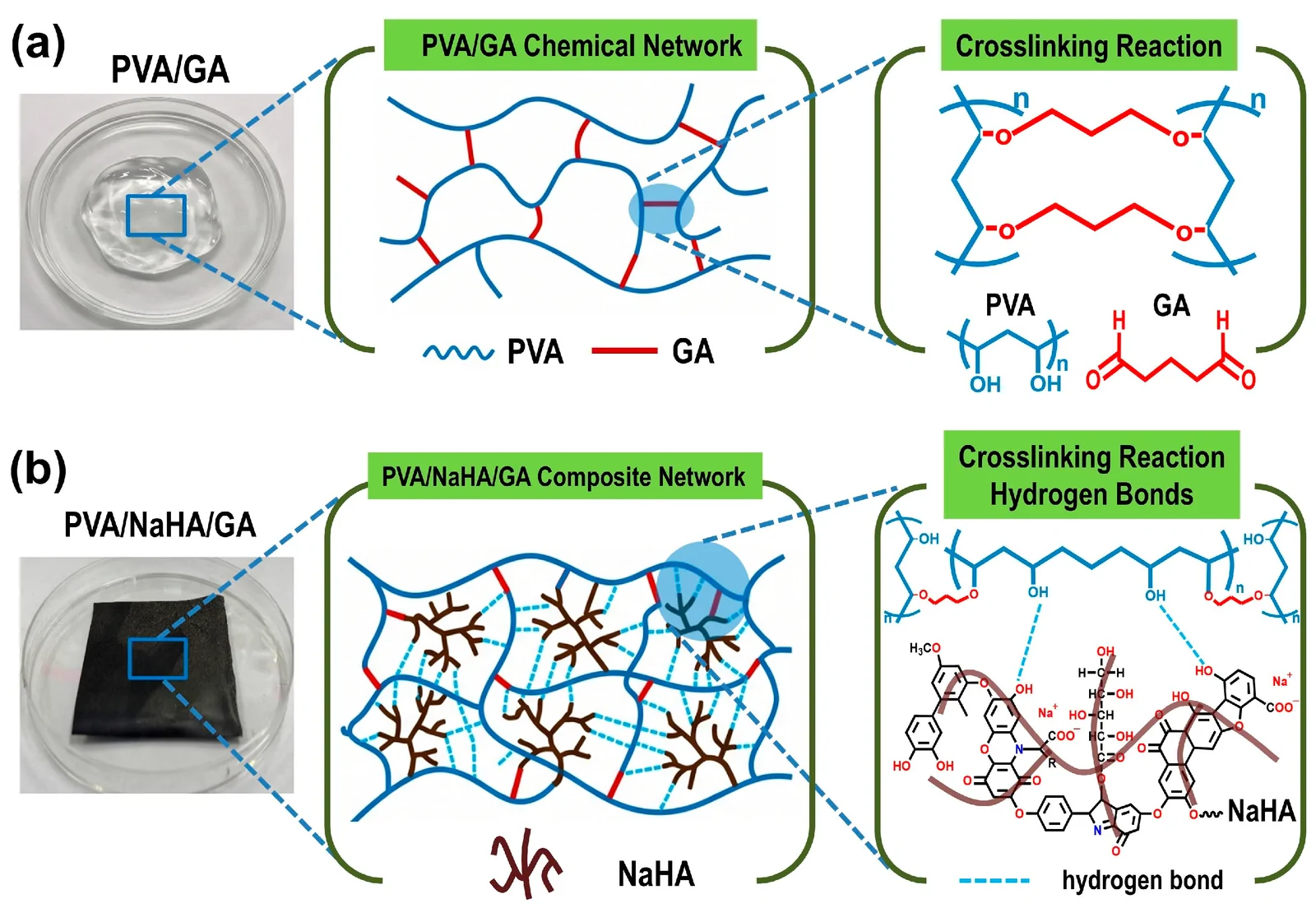
Schematic illustrations of the crosslinked network structures in (**a**) PVA/GA hydrogel and (**b**) PVA/NaHA/GA composite membranes.

Figure 1 presents schematic illustrations of the crosslinked network structure in the PVA/GA and PVA/NaHA/GA systems, respectively. Owing to the abundance of hydroxyl and carboxyl groups within the HA macromolecular backbone, NaHA can establish extensive hydrogen-bonding interactions with PVA chains, thereby reinforcing the polymer network and enhancing the mechanical performance of the resulting composite membranes. In the binary PVA/GA system without NaHA, the aldehyde groups of GA react with the hydroxyl groups of PVA through acetalization, generating a chemically crosslinked network that serves as the primary structural framework. However, the nonuniform distribution of crosslinking sites together with relatively weak intermolecular interactions between polymer chains results in a sparse network with limited energy dissipation capability, making it insufficient to withstand external mechanical deformation effectively (Figure 1a). The incorporation of NaHA fundamentally reconstructs the network architecture. The abundant carboxyl and phenolic hydroxyl groups on the NaHA macromolecules form a dense network of intermolecular and intramolecular hydrogen bonds with the hydroxyl groups of PVA. As illustrated in the enlarged schematic (Figure 1b), in the NaHA-containing membranes, the oxygen-bearing functional groups of NaHA are capable of forming additional hydrogen bonds with PVA, whereas a portion of NaHA may remain physically entrapped within the polymer matrix. Such intermolecular interactions are likely to modulate stress transfer efficiency and thereby give rise to a mechanical response that is dependent on the membrane composition [23].

**Figure 2:**
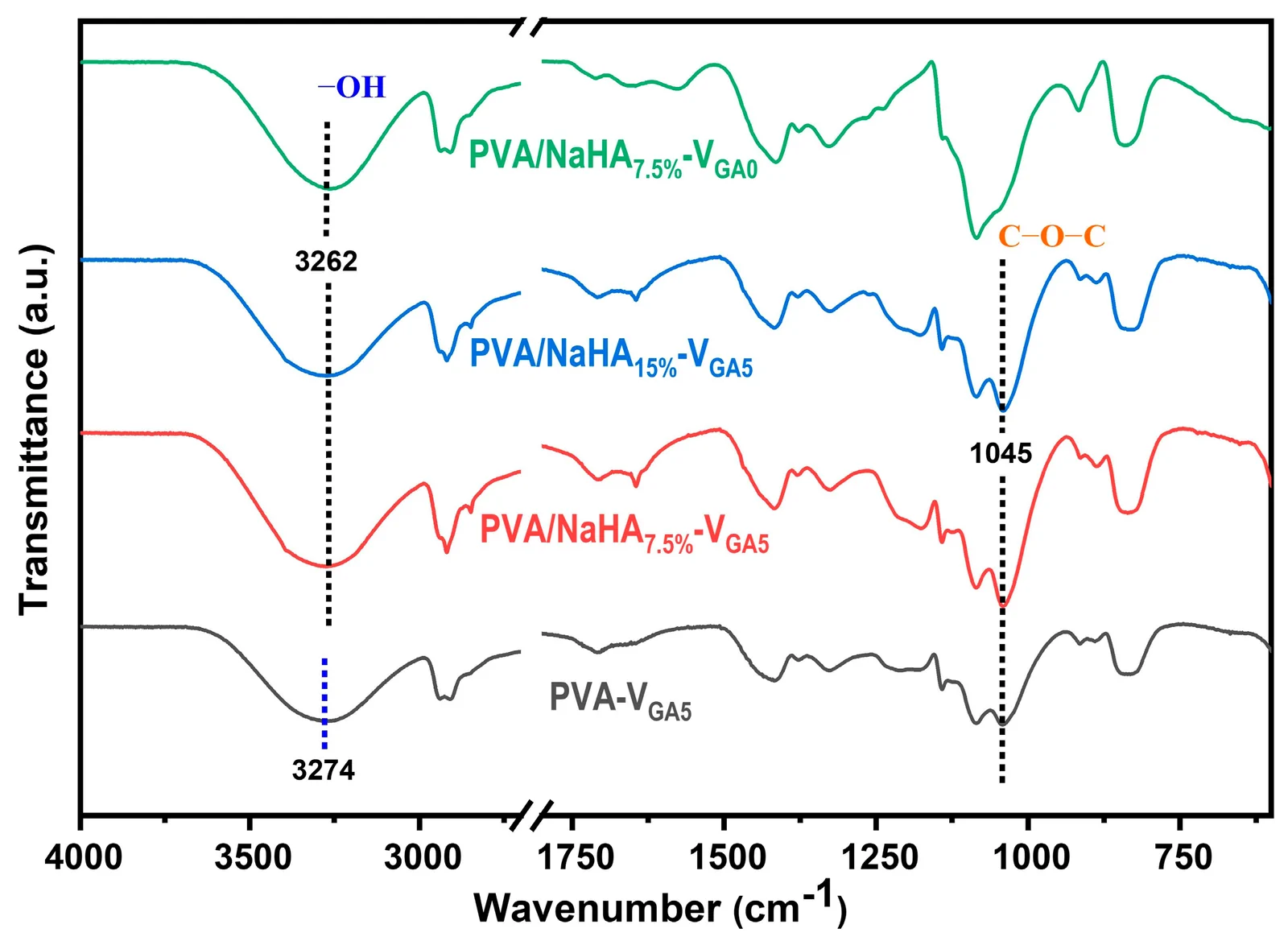
FTIR spectra of PVA-V_GA5_, PVA/NaHA_7.5%_-V_GA0_, PVA/NaHA_7.5%_-V_GA5_, and PVA/NaHA_15%_-V_GA5_ composite membranes.

Figure 2 shows the FTIR spectra of PVA/NaHA/GA composite membranes as a function of NaHA content. As shown in Figure 2, the GA-crosslinked samples exhibit a pronounced absorption band at 1045 cm^−1^, corresponding to the stretching vibration of the C–O–C linkage within the acetal ring formed during the acetalization reaction, confirming the successful formation of the covalent crosslinked framework [38]. Upon the introduction of NaHA, the O-H stretching band broadened and shifted from 3274 cm^−1^ for PVA-V_GA5_ to 3262 cm^−1^ for the NaHA-containing membranes. Such spectral variations are indicative of modifications in the hydrogen-bonding network, suggesting specific interactions between NaHA and the remaining hydroxyl groups of PVA. This shift reflects a redistribution of electron density caused by strong hydrogen-bonding interactions and provides evidence for the formation of extensive intermolecular and intramolecular hydrogen bonds between the abundant oxygen-containing functional groups of NaHA (primarily carboxyl and phenolic hydroxyl groups) and the residual hydroxyl groups of PVA [25].

**Figure 3:**
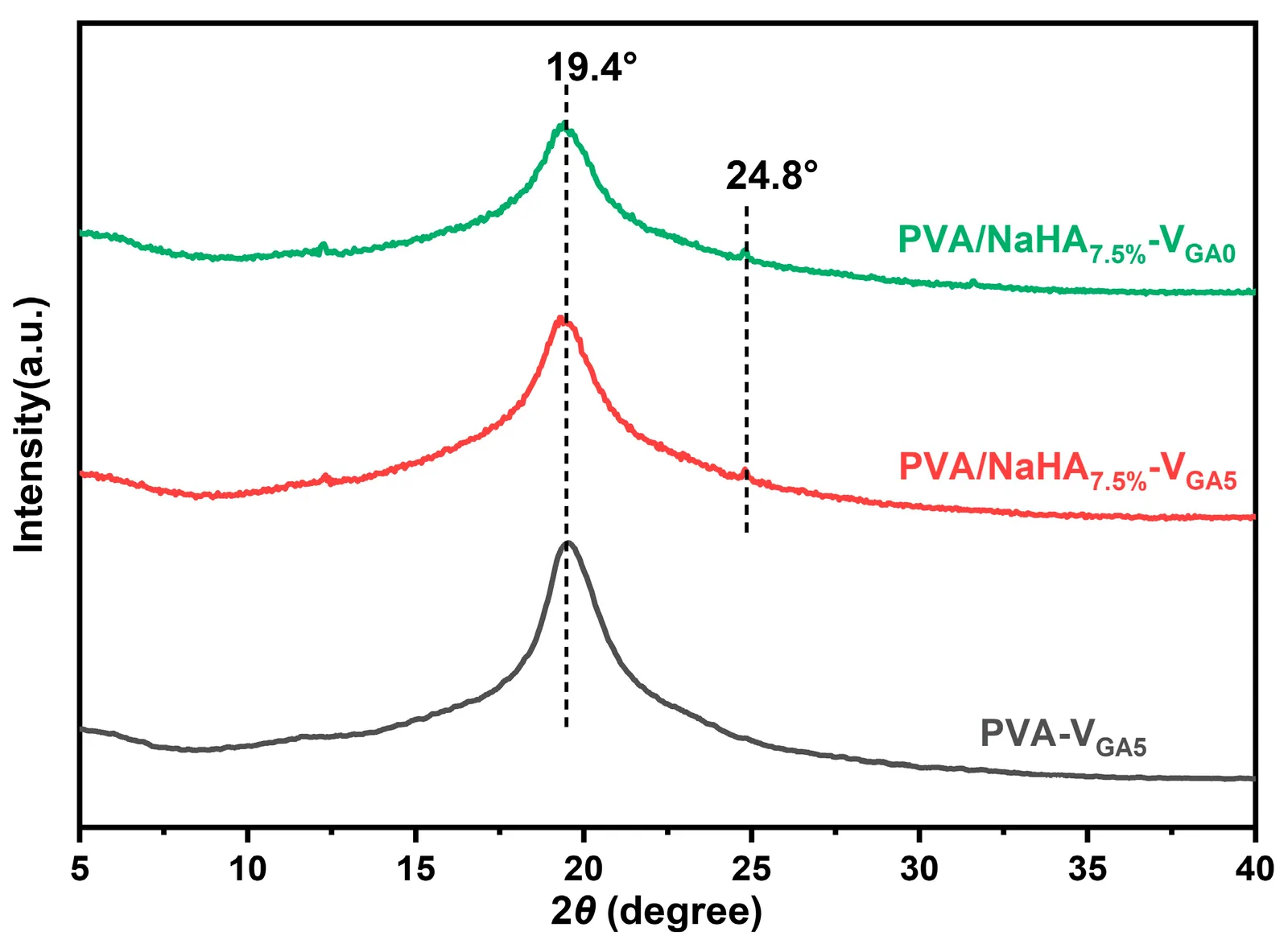
XRD characterizations of PVA-V_GA5_, PVA/NaHA_7.5%_-V_GA0_, and PVA/NaHA_7.5%_-V_GA5_ composite membranes.

The evolution of the crystalline microstructure was further investigated via X-ray diffraction (XRD) (Figure 3). The PVA-V_GA5_ membrane exhibits a broad diffraction peak centered at 2θ ≈ 19.4°, which is characteristic of the semicrystalline structure of PVA originating from intermolecular hydrogen bonding and local chain ordering. Upon incorporation of NaHA, both the physically blended (PVA/NaHA_7.5%_-V_GA0_) and chemically crosslinked (PVA/NaHA_7.5%_-V_GA5_) composite membranes display a marked decrease in the intensity of this diffraction peak accompanied by significant peak broadening. Rigid covalent crosslinking by GA suppresses segmental relaxation of PVA chains, while immobilized NaHA macromolecules disrupt polymer chain packing and crystallization through hydrogen bonding. More importantly, an additional diffraction peak appears at 2θ ≈ 24.8° exclusively in the NaHA-containing samples, while it is entirely absent in the pristine PVA-V_GA5_ membrane. Given the sharpness of this diffraction feature and the aromatic character of NaHA, this peak is tentatively attributed to the formation of nanoscale supramolecular aggregates of NaHA. Owing to steric confinement imposed by the crosslinked network and local heterogeneity in the crosslinking process, a fraction of NaHA molecules fail to be effectively integrated into the extended hydrogen-bonding network of PVA. These observations are consistent with previous reports on the aggregation behavior of humic acid derivatives [39].

Collectively, the FTIR and XRD findings support the formation of a heterogeneous composite structure, consisting of a GA-crosslinked PVA network intertwined with NaHA-rich domains, which are primarily associated via hydrogen-bonding or physical entrapment. This hierarchical architecture provides a structural framework for interpreting and rationally modulating the nonlinear mechanical behavior and fracture characteristics of the composite membranes.

### 3.2. Mechanical Performance of PVA/NaHA/GA Composite Membranes

Previous studies on PVA/NaHA composite membranes have primarily focused on bulk three-dimensional systems fabricated via physical or chemical crosslinking for applications such as heavy-metal adsorption and controlled fertilizer release [23,24,25]. In contrast, this work develops chemically crosslinked PVA/NaHA composite membranes that retain hydrogel characteristics while offering enhanced mechanical strength, water resistance, and structural stability. Considering the prospective applications in agricultural mulch films and controlled-release delivery systems, we systematically investigated the effect of composition on mechanical properties to establish structure–property relationships.

**Figure 4:**
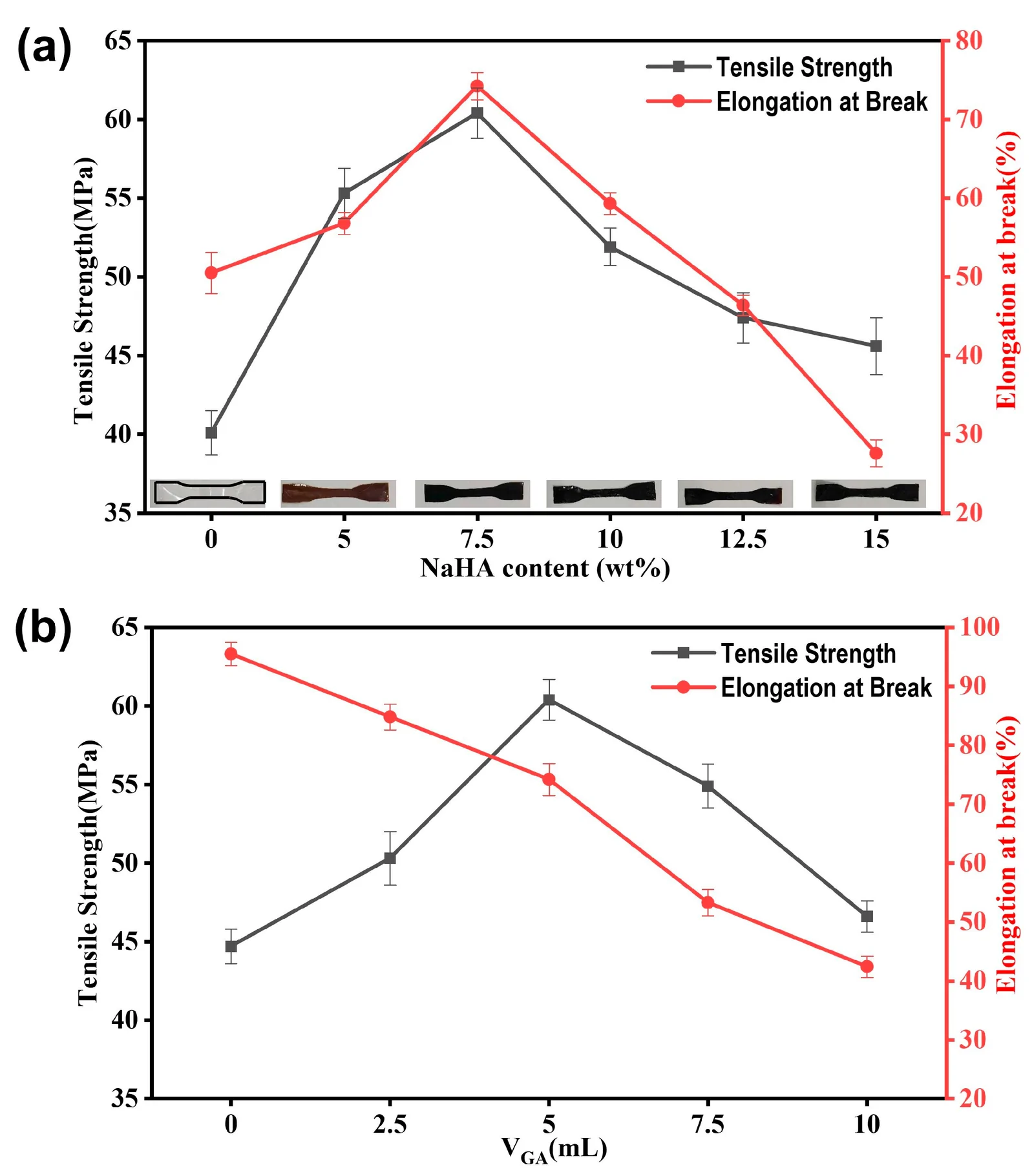
Dependence of tensile strength and elongation at break on (**a**) NaHA content (series 1) and (**b**) GA loading (V_GA_) (series 2).

Figure 4 summarizes the dependence of tensile strength and elongation at break on (a) NaHA content (Series 1) and (b) GA loading volume (Series 2). At a fixed V_GA_ = 5 mL, the pristine PVA-V_GA5_ membrane achieved a tensile strength of 40.1 MPa and an elongation at break of 50.5%. Upon gradual incorporation of NaHA, the mechanical properties evolved in a distinctly non-monotonic manner. The optimal performance was achieved at a NaHA content of 7.5 wt% (PVA/NaHA_7.5%_-V_GA5_), where the tensile strength and elongation at break increased to 60.4 MPa and 74.2%, corresponding to improvements of 50.6% and 46.9%, respectively, compared with the pristine PVA-V_GA5_ membrane (Figure 4a). The simultaneous increase in strength and elongation at intermediate NaHA loadings is attributed to the synergistic effect of uniform dispersion and the formation of a dynamic hydrogen-bonding network. When the NaHA content was further increased to 15 wt%, however, the tensile strength and elongation at break decreased markedly to 45.6 MPa and 27.6%, respectively. This deterioration is caused by excessive NaHA domains aggregating in the PVA matrix due to steric exclusion. These aggregates compromise the continuity of the polymer network and introduce stress-concentrating defects, thereby causing a progressive shift in the fracture mode from ductile to brittle behavior.

To further elucidate the influence of crosslinking density on the mechanical response, the NaHA content was fixed at 7.5 wt%, while the GA loading volume was systematically varied (Figure 4b). The physically blended membrane without chemical crosslinking (PVA/NaHA_7.5%_-V_GA0_) exhibited a high elongation at break of 95.5%, but its tensile strength remained relatively low (44.7 MPa), indicating insufficient structural integrity for practical applications. As the GA loading increased from 2.5 mL to 5.0 mL, the covalent network became progressively more complete, leading to an increase in tensile strength from 50.3 MPa to 60.4 MPa, while maintaining a high elongation at break of 74.2%. These results indicate an excellent balance between strength and toughness. Further increasing the GA loading to 7.5 mL and 10 mL caused a pronounced deterioration in mechanical performance. The tensile strength decreased to 54.9 MPa and 46.6 MPa, while the elongation at break dropped sharply to 53.3% and 42.4%, respectively. Excessive GA promotes rapid local acetalization, generating an overly dense covalent network that severely restricts polymer chain slippage and segmental relaxation. Such over-crosslinking not only immobilizes polymer chains but also suppresses the dynamic energy-dissipation capability of the hydrogen-bonding network during deformation, thereby increasing structural heterogeneity and generating localized brittle regions that ultimately lead to premature brittle fracture at relatively low strain [40].

**Figure 5:**
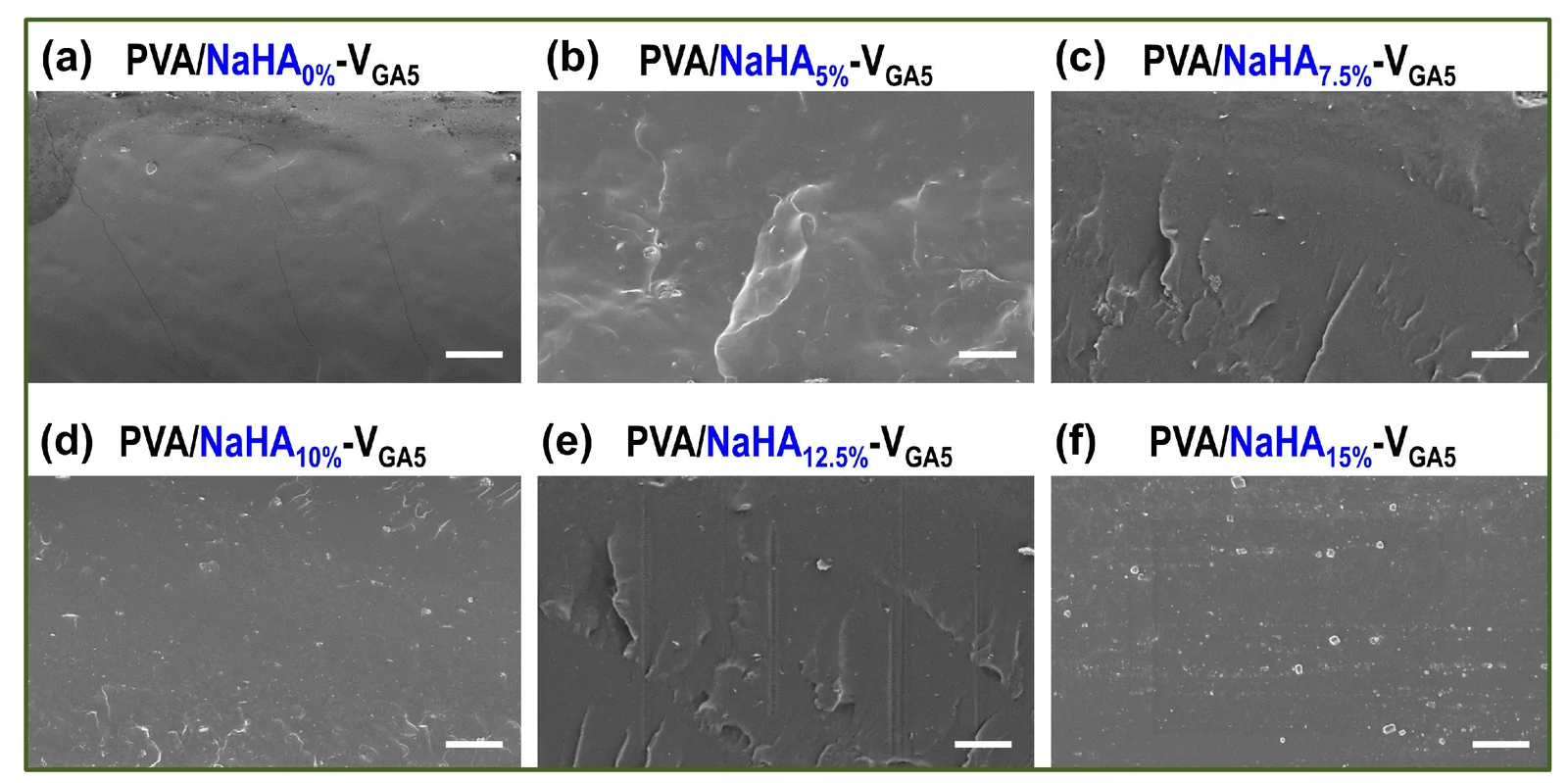
Cross-sectional SEM micrographs showing the microstructures of a series of composite membranes with different NaHA contents (series 1): (**a**) PVA/NaHA_0%_-V_GA5_, (**b**) PVA/NaHA_5%_-V_GA5_, (**c**) PVA/NaHA_7.5%_-V_GA5_, (**d**) PVA/NaHA_10%_-V_GA5_, (**e**) PVA/NaHA_12.5%_-V_GA5_, and (**f**) PVA/NaHA_15%_-V_GA5_. Scale bar: 5 μm.

To correlate the mechanical behavior with the underlying microstructure, the cryo-fracture surfaces of the composite membranes with different NaHA contents were examined by means of scanning electron microscopy (Figure 5). The pristine PVA-V_GA5_ membrane exhibited a dense and smooth fracture surface with a characteristic amorphous morphology, indicating the formation of a homogeneous chemically crosslinked network (Figure 5a). At NaHA contents of 5 wt% and 7.5 wt%, no obvious phase separation or isolated particles were observed. Instead, the fracture surfaces displayed rough, river-like ductile fracture patterns (Figure 5b,c), suggesting excellent interfacial compatibility between NaHA and the PVA matrix. Through extensive hydrogen-bonding interactions, NaHA is uniformly anchored within the network and serves as an effective stress-transfer center, promoting localized plastic deformation and enhancing fracture-energy dissipation. In contrast, when the NaHA content exceeded 10 wt%, the fracture morphology deteriorated markedly (Figure 5d–f). In particular, membranes with 12.5 wt% and 15 wt% NaHA showed numerous micron-sized aggregates. These aggregates, together with the associated microcracks, disrupted the continuity of the polymer matrix, acted as stress-concentration sites, and interrupted efficient stress transfer, ultimately causing brittle failure at relatively low strain. The formation of the aforementioned structural features corresponds to the reduction in tensile strength and elongation at break observed with increasing NaHA content, as shown in Figure 4a. Such structural evolution is likely to promote localized stress concentrations, thereby contributing to the deterioration of mechanical performance.

**Figure 6:**
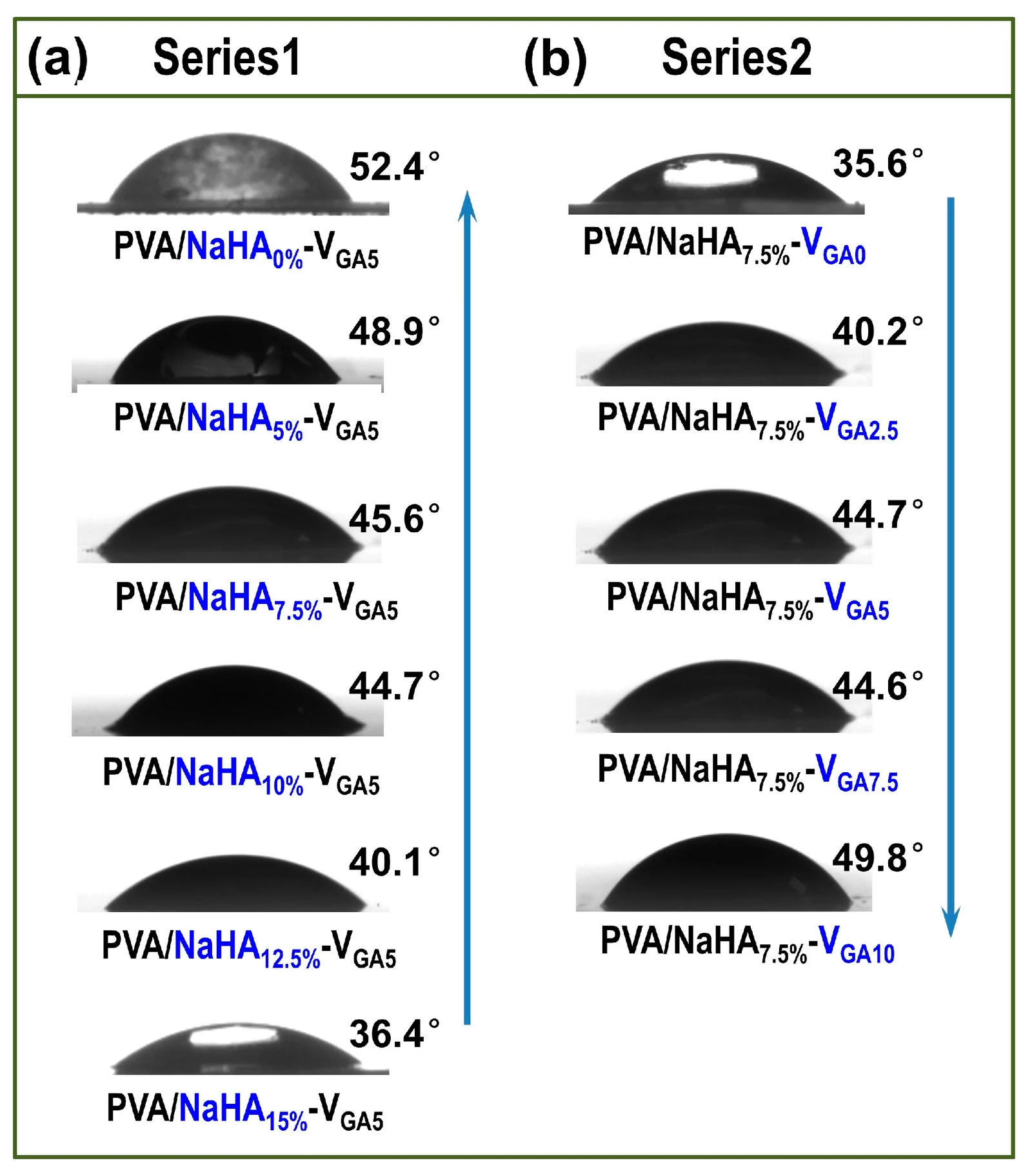
Static water contact angle (WCA) evolution of a series of composite membranes. (**a**) WCA as a function of NaHA content at a fixed GA loading (V_GA_ = 5 mL) (series 1). (**b**) WCA dependence on GA loading with a fixed NaHA content of 7.5 wt% (series 2).

The surface wettability of the composite membranes is indicative of both their chemical composition and surface organizational structure [41]. Figure 6a presents the variation in static water contact angle as a function of NaHA content. The pristine PVA-V_GA5_ membrane exhibited a contact angle of 52.4°, indicating a relatively hydrophobic surface. As the NaHA content increased to 15 wt%, the contact angle continuously decreased to 36.4°, demonstrating a significant increase in surface hydrophilicity. This trend may be attributed to the abundant oxygen-containing polar groups, particularly carboxyl and phenolic hydroxyl groups, present on the NaHA macromolecules, which enhance hydration and reduce the solid–air interfacial tension. Conversely, chemical crosslinking exerted an opposite effect on surface wettability. As shown in Figure 6b, increasing the GA loading volume progressively increased the contact angle from 35.6° for the uncrosslinked PVA/NaHA_7.5%_-V_GA0_ membrane to 49.8°. This increase in hydrophobicity reflects the consumption of hydrophilic hydroxyl groups of PVA during acetalization, along with the formation of a dense covalent network that reduces free volume and restricts water permeation. By balancing the NaHA content and the crosslinking density, the optimized PVA/NaHA_7.5%_-V_GA5_ membrane exhibited a moderate water contact angle of approximately 45°. This intermediate wettability represents a desirable balance between hydrophilicity and water resistance. Compared with the uncrosslinked membrane, which undergoes excessive hydration and swelling, the chemically crosslinked network effectively suppresses excessive water uptake, thereby maintaining dimensional stability and mechanical integrity in aqueous environments. At the same time, sufficient surface hydrophilicity is retained to promote favorable wetting and liquid spreading, providing an appropriate interfacial environment for controlled water transport and subsequent release of the encapsulated functional components [42].

### 3.3. In Vitro Sustained-Release Profiles of PVA/NaHA/GA Composite Membranes

**Figure 7:**
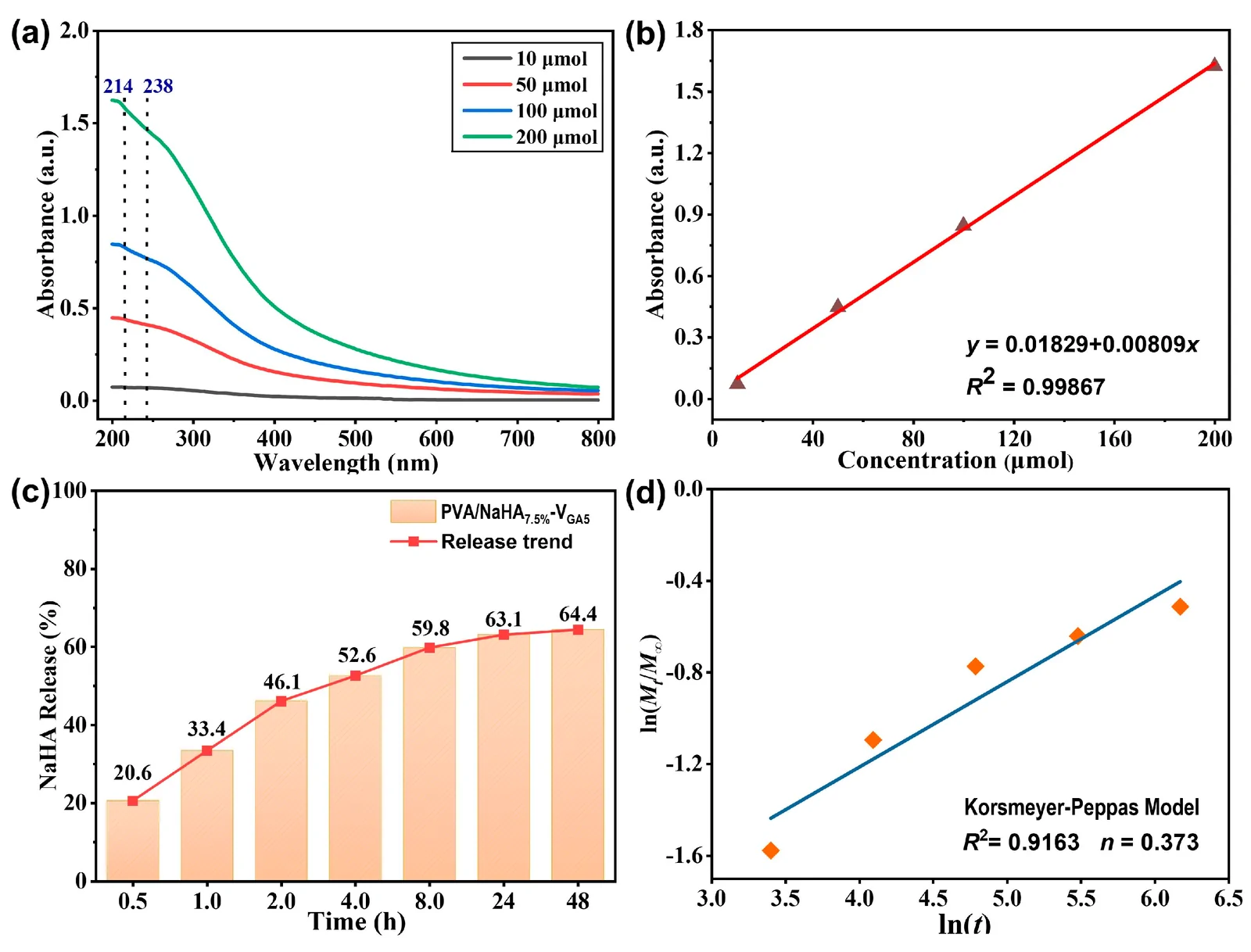
NaHA release kinetics and mathematical modeling of PVA/NaHA_7.5%_-V_GA5_ composite membrane: (**a**) UV-Vis absorption spectra at different concentrations. (**b**) Linear fitting curve of absorbance intensity at an average absorbance (214~238 nm) as a function of concentration. (**c**) Cumulative release profile of NaHA over 48 h. (**d**) Linear fitting curve based on the Korsmeyer–Peppas kinetic model.

Figure 7 illustrates the release kinetics of NaHA from the PVA/NaHA_7.5%_-V_GA5_ composite membranes. To minimize optical interference during the determination of the released NaHA concentration, the UV–Vis calibration curve was constructed by averaging the absorbance over the broadened absorption band between 214 and 238 nm (Figure 7a,b). The cumulative release profile of the optimized membrane (PVA/NaHA_7.5%_-V_GA5_) over 48 h is presented in Figure 7c. During the initial release stage (0~1 h), an apparent burst release of approximately 33.4% was observed, which is attributed to the rapid dissolution of NaHA macromolecules weakly associated with the surface network through hydrogen-bonding interactions. As the release proceeded, water penetration into the densely crosslinked polymer network became increasingly restricted, leading to a pronounced reduction in the release rate and the establishment of a sustained-release regime. The cumulative release reached 59.8% after 8 h and 64.4% after 48 h, demonstrating that the covalently crosslinked network effectively confined the encapsulated NaHA and provided prolonged release behavior.

To further elucidate the release mechanism, the experimental data were fitted using the zero-order, first-order, Higuchi, and Korsmeyer–Peppas kinetic models (Table 2) [43,44]. According to the applicability criterion of the Korsmeyer–Peppas model, only the initial release stage with a cumulative release fraction below 60% (*Mt/M∞* ≤ 0.6, corresponding to the first 8 h) was used for regression analysis to ensure reliable determination of the kinetic parameters. The release profiles showed poor agreement with the zero-order and first-order models, whereas significantly better correlations were obtained with the Higuchi model (*R*^2^ = 0.8820) and the Korsmeyer–Peppas model (*R*^2^ = 0.9163) (Figure 7d). Fitting the Korsmeyer–Peppas equation (*Mt/M∞* = *kt^n^*) yielded a release exponent of *n* = 0.373, which falls within the pseudo-Fickian diffusion regime (*n* ≤ 0.45) for a planar film geometry, suggesting that diffusion may be the predominant mechanism under the tested conditions. However, due to the limited number of early-time data points, the use of linearized *R*^2^ values without comparative nonlinear model fitting, and the absence of residual-based diagnostics, a definitive assignment of the release mechanism cannot be established from the current dataset. The 48 h time point is therefore reported as the final experimental observation rather than as a confirmed equilibrium release level.

Based on the Flory–Rehner equilibrium swelling theory, the effective crosslinking density (*ν*_e_) of the PVA/NaHA_7.5%_-V_GA5_ composite membrane was estimated as approximately 4.97 × 10^−5^ mol/cm^3^. Under the adopted assumptions, this corresponds to an average effective mesh size (ξ) on the order of tens of nanometres, with a calculated value of roughly 33 nm [26]. It must be emphasized, however, that this estimate represents a model-dependent average derived from ideal-network theory and should not be interpreted as a directly measured pore dimension. Previous studies have shown that humic acid can spontaneously self-assemble into supramolecular micelle-like aggregates with characteristic diameters ranging from 30 to 120 nm in aqueous solution [45,46]. Combining this size relationship with the experimentally observed burst release (33.4%) followed by sustained release (64.4%), this correlation implies that a considerable portion of NaHA is not molecularly dispersed within the PVA network but rather exists as encapsulated supramolecular aggregates. When the network mesh size becomes comparable to the dimensions of these aggregates, steric exclusion and hydrogen-bonding interactions synergistically suppress the outward diffusion of NaHA trapped within the interior of the membrane. Consequently, the system exhibits a heterogeneous release behavior characterized by rapid desorption of surface-associated NaHA, along with diffusion-limited transport of aggregate-encapsulated NaHA from the interior. This dual-mode transport is thus considered the primary factor governing the sustained release performance of the composite membrane system.

**Figure 8:**
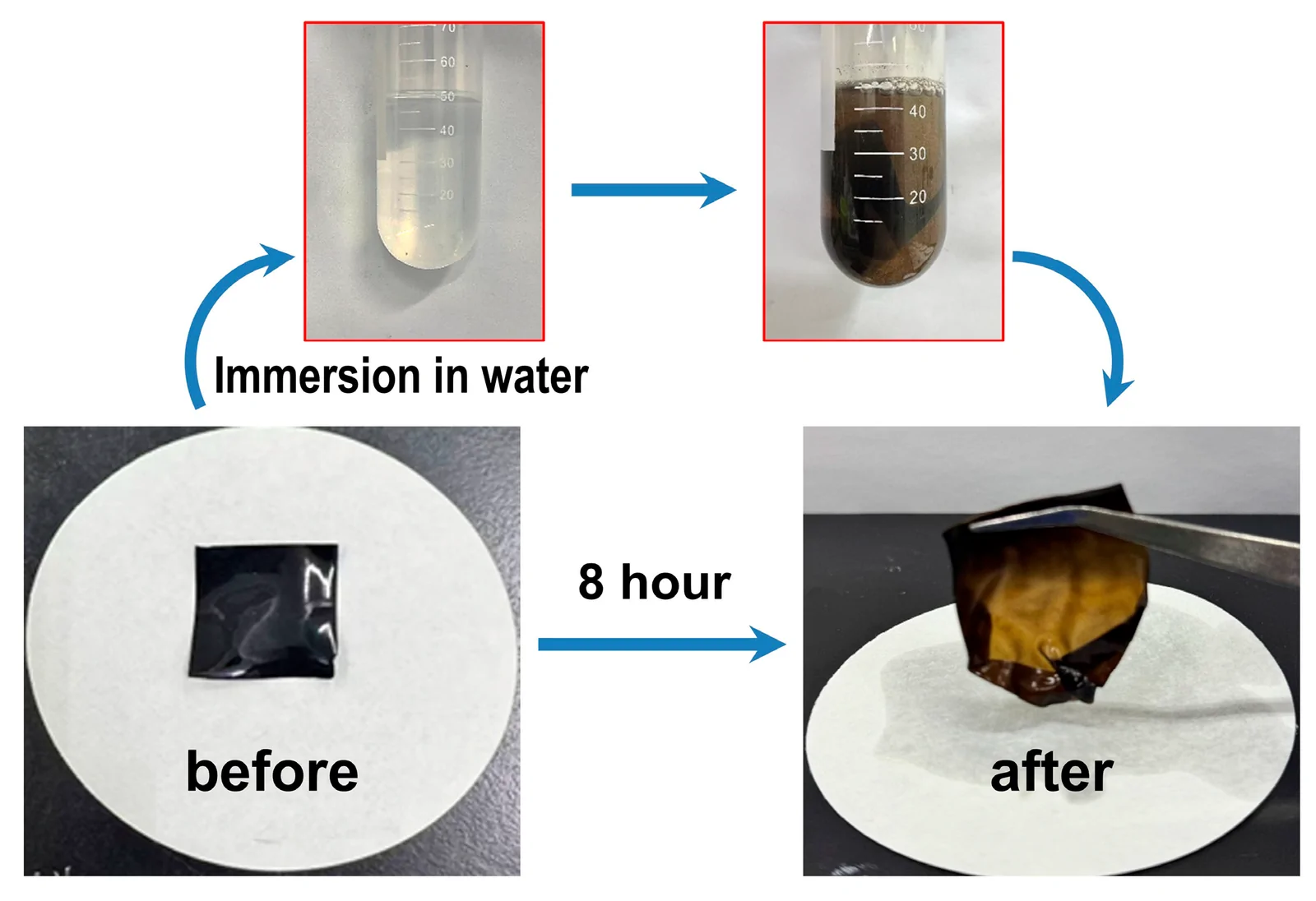
Macroscopic visual evidence of the sustained release and structural stability of the optimized composite membrane before and after 8 h of continuous water immersion.

Figure 8 presents macroscopic visual evidence of the sustained release and structural stability of the optimized PVA/NaHA_7.5%_-V_GA5_ composite membrane before and after 8 h of continuous water immersion. As free and weakly bound NaHA macromolecules gradually diffused into the surrounding solution, the immersion medium changed from colorless to dark brown, while the membrane itself became progressively more transparent, providing visual evidence of the internal mass-transfer process. More importantly, despite prolonged exposure to water, the composite membrane maintained excellent structural integrity without noticeable swelling, disintegration, or fracture. These observations are consistent with the gradual cumulative release profile and demonstrate that the rigid covalent network effectively overcomes the poor aqueous stability typically associated with hydrophilic polymer materials. Therefore, mass transport within the membrane remains predominantly diffusion-controlled, confirming that the crosslinking density plays a decisive role in regulating diffusion pathways. These findings provide both structural insight and experimental evidence supporting the potential application of the PVA/NaHA/GA composite membrane as a controlled-release carrier for agricultural nutrients and as a functional liquid mulch material. It should be noted, however, that residual glutaraldehyde content and leachate toxicity were not addressed in the present study; these parameters will require systematic evaluation prior to any direct environmental application.

## 4. Conclusions

The crosslinked PVA/NaHA/GA composite membranes were systematically investigated with respect to the effects of NaHA content and GA loading on their structural and functional properties. The incorporation of NaHA into the PVA matrix promotes the formation of self-assembled composite architectures that effectively reconcile structural integrity with enhanced mechanical performance. In this system, tensile strength is predominantly governed by the crosslinking density, while elongation at break is largely dictated by the plasticizing and energy-dissipation mechanisms imparted by NaHA. In vitro release studies indicate that the sustained-release behavior is co-regulated by steric hindrance from the network mesh size and hydrogen-bonding interactions, resulting in a biphasic release profile characterized by an initial burst phase followed by a prolonged diffusion-dominated stage. More importantly, this molecular design strategy integrates naturally heterogeneous humic macromolecules into chemically crosslinked polymer networks, thereby providing new insights into the relationships among network topology, mass transport, and mechanical performance. The proposed design concept may also be extended to other biomass-derived macromolecules for the development of sustainable multifunctional polymer materials.

## Figures and Tables

**Table 1:** Composition and designation of PVA/NaHA/GA composite membranes.

	Sample Code	PVA (g)	NaHA (g)	NaHA (wt%) ^1^	V_GA_ (mL) ^2^
Series 1 NaHA content variation	PVA-V_GA5_	5	0	0	5 mL
	PVA/NaHA_5.0%_-V_GA5_	5	0.25	5	5 mL
	PVA/NaHA_7.5%_-V_GA5_	5	0.375	7.5	5 mL
	PVA/NaHA_10%_-V_GA5_	5	0.5	10	5 mL
	PVA/NaHA_12.5%_-V_GA5_	5	0.625	12.5	5 mL
	PVA/NaHA_15%_-V_GA5_	5	0.75	15	5 mL
Series 2 GA loading volume variation	PVA/NaHA_7.5%_-V_GA0_	5	0.375	7.5	0
	PVA/NaHA_7.5%_-V_GA2.5_	5	0.375	7.5	2.5 mL
	PVA/NaHA_7.5%_-V_GA7.5_	5	0.375	7.5	7.5 mL
	PVA/NaHA_7.5%_-V_GA10_	5	0.375	7.5	10 mL

^1^ The weight percentage of NaHA is calculated based on the dry mass of PVA. ^2^ V_GA_ denotes the loading volume of the 5.0 wt% GA working solution required for each crosslinking reaction.

**Table 2:** Kinetic parameters of NaHA release calculated using the mathematical models ^1^.

Kinetic Model	Mathematical Equation	*R* ^2^	*k*	*n*
Korsmeyer–Peppas	*M_t_*/*M_∞_* = *kt^n^*	0.9163	0.0667	0.373
Higuchi	*M_t_*/*M_∞_* = *k_H_t*^1/2^	0.8820	0.0274	—
First-order	*ln*(1 − *M_t_*/*M_∞_*) = −*k*_1_*t*	0.8335	0.0019	—
Zero-order	*M_t_*/*M_∞_* = *k*_0_*t*	0.7580	0.0012	—

^1^ *M_t_/M_∞_* represents the fractional release of NaHA at time *t* (min). *R*^2^ is the coefficient of determination. *k* denotes the kinetic release rate constant specific to each model. *n* is the diffusional exponent from the Korsmeyer–Peppas model, indicative of the NaHA release mechanism.

## Data Availability

The original contributions presented in this study are included in the article. Further inquiries can be directed to the corresponding authors.
